# Preferring self-management behavior of patients with chronic kidney disease

**DOI:** 10.3389/fpubh.2022.973488

**Published:** 2022-12-02

**Authors:** Xiaoli He, Yu Wang, Chenchen Feng, Le Luo, Usama Khaliq, Faheem Ur Rehman, Xinli Zhang

**Affiliations:** ^1^West China School of Nursing/Department of Outpatient, West China Hospital, Sichuan University, Chengdu, China; ^2^Department of Industry Engineering and Management, Business School, Sichuan University, Chengdu, China; ^3^School of Civil Engineering and Geomatics, Southwest Petroleum University, Chengdu, China; ^4^Business School, NingboTech University, Ningbo, China

**Keywords:** self-management behavior, principal component analysis, *F*-test, healthcare, CKD

## Abstract

This study explores the preferred behavior of self-management among chronic kidney disease (CKD) patients and offers suggestions for different patients from personalized medicine. According to some related references, a questionnaire was designed in 2020 to collect data from 131 patients with CKD in a general hospital. The Sampling patients showed no difference in their disease progress. The questionnaire covered two aspects of demographic and behavior with 29 items on six dimensions. Statistical methods such as a descriptive analysis of the F test in behavior dimensions on demographic characteristics and Principal component analysis from items have been applied to classify some kinds of self-management behavior into different groups. In the demographic insight, employment status closely relates to self-management behavior, and income is insignificant. In the behavior aspects, according to some key items, we found four types of self–management behavior preferred in the sorting: cognitive-knowledge, Diet-exercise-medical, emotion management, and exercise-medical, which were defined by behavior dimensions. Although patients had the same disease progress, their self-management behavior mainly existed in four types based on critical factors. According to their favorite behavior and personality group, healthcare stakeholders can offer lean support for improving patients' self-management of CKD in China.

## Introduction

Chronic kidney disease (CKD) is a common lifelong disease in clinical science. It is prolonged and debilitating (1), posing a significant threat to public health. It has been estimated that there are more than 70 million CKD patients globally (2). According to the World Health Organization, 14 out of every 100,000 people will die from CKD in 2030. Patients with CKD must self-manage their illness to a large extent to slow disease progression (3). Lean implementation of chronic disease management (CDM) significantly benefits individuals, society, and the economy (4).

In general, there are four CDM models centered on CDM. Stanford model improves patient self-efficacy through a chronic disease self-management program (CDSMP) (5). The Flinders Model (FM) proposes that a patient is the ultimate decision-maker and emphasizes that doctors and patients make joint decisions and share responsibilities (6). Expert Patients Program (EPP) emphasizes that patients participate in medical decision-making and any self-management project to establish continuous feedback, evaluation, and assessment mechanism (7). The Chronic Care Model (CCM) emphasizes that patients should be involved in medical decision-making (8). The above CDM models show that patient self-management behaviors are crucial in optimizing diagnosis and treatment plans, ensuring patients' safety, and improving patient compliance with treatment (9). Self-management is generally defined as the daily management of chronic conditions by individuals during a period of the disease (10).

Although self-management behavior is a complex endeavor, which is influenced by the stage of the disease (11), demographic characteristics (12), and so on, with the joint participation of both medical staff and patients, it centers on the patient's sense of responsibility for their health, and requires the patient's adjustment and to control their behaviors (13), including beneficial behaviors such as daily diet, physical exercises, mental health, and disease treatment (14). Finally, it leads to a higher quality of life (15). Studies reported that the multiple self-management strategies adopted by chronic disease patients eventually follow a specific trend and pattern. These trends and patterns affect chronic disease progression (9). Patterns have certain differences (16). This is conducive to eliminating waste and improving medical efficiency and quality (17). Therefore, from the perspective of the influencing factors of self-management, self-management is not that patient finishing every management works by themselves, which need some lean support from the related stakeholders, such as a doctor, family, and society environment. So what kind of support should they offer from a broader?

This study identifies patient self-management behavior patterns to establish systematic lean healthcare support from the related stakeholders for chronic disease patients using data from patients' surveys. In the methodology, Behavior pattern recognition is to distinguish behavior differences from the environment and make decisions for different types of people (18), which aims to extract information features and classify them into different groups (19). In general, behaviors often have independent multi-attribute features; therefore, most studies use principal component analysis to reduce the dimensionality to extract key features (20) and recognize patterns (21). This study applied the Test and Principal component method to study Self-management behavior using the actual data survey of chronic disease patients.

The following contents of this study are organized as follows: Section Methods introduces methods, Section Results shows results, Section Discussion is the discussion, and Section Conclusions offers conclusions.

## Methods

### Study design

This study used purposive sampling to enroll subject patients diagnosed with CKD in a general hospital in southwest China for one month. All of the included patients came from the CKD outpatient department. The estimated sample size in the empirical case was more than 100 subjects who could answer the survey questions face to face. Another inclusion criterion is that these outpatients had no disease progression, which can be a control variable to reveal some effective self-management behavior in the same disease group. This study enrolled the sample size of subjects to be not less than 130 subjects, thinking about a 30% questionnaire loss rate.

### Behavior instrument

Most studies constructed an assessment scale for chronic disease self-management behavior based on some relevant theories in chronic disease self-management. Four self-management models, as mentioned before, put forward that different chronic diseases need to design with specific survey scales (22). The first part of the questionnaire is demographic characteristics designed for six items. The second part is based on the three major tasks of chronic disease self-management as daily life management, medical management of diseases, and emotional and cognitive management (23). This study designed six behavioral dimensions including 29 items, namely treatment management with five items, exercise behaviors with four items, diet behaviors with five items, emotional management with five items, disease cognition with five items, and self-management knowledge with five items. The questionnaire was scored using a five-point Likert scale, and each item was evaluated on an agreement scale of 1–5. Higher scores indicate preferring the self-management behavior of patients. These 29 items are all categorical variables, as shown in Table 1. The score variables of the six behavior dimensions are *y*_*ij*_ (*i, j*ε(1…,6)).

**Table 1:** Questionnaire dimensions and items.

**Dimensions**	**Questionnaire items**	**Variables**
Medical management	Follow the doctor's advice to take medications and perform various treatments on time	y_11_
	Strictly record blood pressure, weight, and other indicators every day	y_12_
	Do not take non-doctoral drugs on your own	y_13_
	Disinfect the room on time, maintain ventilation, and actively eliminate infection factors (to prevent respiratory, urethral, skin infections, etc.)	y_14_
	Report abnormal symptoms to the doctor or nurse promptly	y_15_
Exercise behaviors	Appropriate aerobic exercise (3 times/week, 30min/time), walking, cycling, Tai Chi, etc.	y_21_
	Do not engage in strenuous exercises such as playing ball and riot walk	y_22_
	Live and rest according to the schedule	y_23_
	Self-massage regularly	y_24_
Diet behaviors	Eat low-salt, less fried foods, and fresh vegetables	y_31_
	Follow the doctor's advice to eat quantitative protein and calcium	y_32_
	Quit smoking, alcohol, coffee, and spicy food	y_33_
	Maintain clean eating habits	y_34_
	Regular and quantitative eating	y_35_
Emotional management	Anxiety, irritability, depression, and other adverse symptoms	y_41_
	Happy and cheerful	y_42_
	Talk to others when you are troubled	y_43_
	Establish a variety of hobbies and interests	y_44_
	Proactively resolve conflicts between self and others	y_45_
Disease cognition	Typical symptoms of chronic kidney disease in the early, middle, and late stages	y_51_
	The various hazards of chronic kidney disease	y_52_
	Various common treatments for chronic kidney disease	y_53_
	Common drugs and adverse reactions to chronic kidney disease	y_54_
	Normal values of various biochemical indexes of chronic kidney disease	y_55_
	Chronic Kidney Disease Complications	y_56_
Self-management knowledge	The importance of compliance	y_61_
	The importance of self-management behavior	y_62_
	Knowledge of diet, exercise, and lifestyle habits	y_63_
	Methods of regulating adverse psychological reactions	y_64_

### Ethical considerations

This study was reviewed and approved by the ethics review committees of West China Hospital (2020-740). All participants provided informed consent before participation in the study. The research purposes and procedures were explained to each subject, and before the informed consent form was signed, each subject was informed that they could withdraw from the study at any time and that their rights would not be affected. They were also informed that the data obtained from the questionnaires would be provided only for educational use and would not be disclosed after the study was completed.

### Data analysis

A total of 131 valid questionnaires were distributed. The Cronbach's coefficient of each dimension of the questionnaire is greater than 0.7 databases, with high internal consistency. KMO values of all dimensions are greater than 0.7, with high validity. The encoded data were calculated and analyzed using the software SPSS 26.0. The statistical analyses included descriptive statistics, *F-*test, and principal component analysis for multiple variables. The *F*-test method was used to discriminate differences in self-management behavior dimension on demographic characteristics, which are control variables. α = 0.05 is the test level, and the *P*-values are two-sided probabilities.

## Results

### Demographic

The demographic information characteristics of the sample patients are shown in Table 2. The F test for six behavior dimensions on six characteristics can be seen in the Appendix on gender, education level, employment status, disease expenditure, household income, and age. The whole F test results show in Table 3 between demographic and behavior.

**Table 2:** Demographic information of CKD patients (*n* = 131).

**Demographic characteristics**	**Number**	**Percentage/%**
**Gender**		
Male Female	62 69	47.33 52.67
**Age (years)**		
35> [35,55] >55	50 67 14	38.17 51.15 10.69
**Employment status**		
Yes No	85 46	64.89 35.11
**Education level** Junior high school and below High school or secondary school University and above	22 41 68	16.79 31.30 51.91
**Household income (RMB/month**)		
5,000 < [5,000, 10,000] >10,000	9 71 51	6.87 54.20 38.93
**Disease expenditure (RMB/month**)		
1,000 < [1,000, 2,000] >2,000	31 78 22	23.66 59.54 16.79

**Table 3:** All *F*-test results.

**Dimensions**	**Demographic characteristics**					
	**Gender**	**Education**	**Employment status**	**Disease expenditure**	**Income**	**Age**
Medical management			*			
Exercise behaviors			*			
Diet behaviors			*			
Emotional management		*		*		
Disease cognition			*	*		*
Self-management knowledge			*			

^*^Means significant.

According to Table 3 and the Appendix Tables, from the demographic insight, gender and income are not statistically significant for self-management, while educational background, employment status, disease expenditure, and age have significant differences, though the impact is uneven. Employment status substantially affects the five dimensions other than emotional management, and the patient's educational background and disease expenditure impact emotional management. The higher the education level and the less disease expenditure, the better the emotional management of patients. In the dimension of disease cognition, the patient's age, employment status, and disease expenditure are impact factors. Further analysis found that the poorer the patient's employment, the younger the patient, and the less the disease expenditure, the lower the patient's cognition of the disease. On the contrary, unemployed patients, middle education and middle age, have a high score in self-management, which is similar to Audulv's study (24).

### Principal component

With principal component analysis of 29 items, five principal item components with eigenvalues >1 were obtained, which can explain 71.803% of the original information cumulatively (see Table 4). The explanation level of the fifth principal component is 3.775%, which is < 5%. The first four principal components are finally extracted, and the interpretation degree is 68%. The explanatory variables (items) are sorted according to the descending order of the factor load of 4 principal components in Table 5. According to the 4 principal components, the corresponding self-management pattern can be summarized. See Table 6 for principal components, explanatory variables, and patterns.

**Table 4:** Initial eigenvalues and variance.

**Composition**	**Eigenvalues**	**Variance**	**Cumulative**
		**percentage**	**value %**
F1	14.636	50.470	50.470
F2	1.836	6.331	56.800
F3	1.724	5.943	62.744
F4	1.533	5.285	68.028
F5	1.095	3.775	71.803

**Table 5:** Principal component and factor (item) load matrix.

**Item**	**F1**	**Item**	**F2**	**Item**	**F3**	**Item**	**F4**
y_53_	0.820	y_34_	0.731	y_45_	0.818	y_24_	0.824
y_55_	0.801	y_31_	0.690	y_44_	0.812	y_15_	0.675
y_54_	0.789	y_11_	0.679	y_42_	0.782	y_12_	0.502
y_56_	0.774	y_33_	0.655	y_43_	0.718		
y_52_	0.763	y_35_	0.641	y_41_	0.666		
y_63_	0.651	y_32_	0.629				
y_62_	0.638	y_23_	0.606				
y_61_	0.637	y_22_	0.579				
y_51_	0.635	y_13_	0.551				
y_64_	0.585	y_15_	0.546				

**Table 6:** Principal components, items, and patterns.

**Principal component**	**Items**	**Related dimensions**	**Self-management pattern**
*F*_1_	y_51_, y_52_, y_53_, y_54_, y_55_ y_56_, y_61_, y_62_, y_63_, y_64_	5,6	Cognition-knowledge
*F*_2_	y_31_, y_32_, y_33_, y_34_, y_35_ y_22_, y_23_, y_11_, y_13_, y_15_	3,2,1	Diet-exercise-medicine
*F*_3_	y_41_, y_42_, y_43_, y_44_, y_45_	4	Emotion
*F*_4_	y_21_, y_24_, y_12_	2,1	Exercise-medicine

According to the eigenvalues of the 4 principal components in Table 6, *F*_1_ is the highest one which means disease Cognition-Knowledge pattern dominates. The component loads show that *F*_2_ has a strong positive correlation with ten items in the dimensions of diet, exercise, and medicine management. The eigenvalues of *F*_2_,*F*_3_ and *F*_4_ decrease in order; that is, among the self-management patterns of CKD patients, the preference for the Diet-Exercise-Medicine pattern, Emotion pattern, and Exercise-Medicine pattern decreases in order.

In the self-management pattern, exercise and medicine appeared two times among the four patterns, so it is the primary behavior that leads patients to self-management. Diet behaviors, emotional management, disease cognition, and self-management knowledge only appeared once in each self-management pattern.

According to the principal component analysis results, there is a progressive relationship among the four self-management patterns. Firstly, to improve patients' self-management behavior, patients' cognition of the disease and related knowledge of self-management should be strengthened so that patients understand the disease's causes and can better cooperate with the doctor's advice. After certain basic cognition, patients should be further told to control their diets, exercise appropriately, and cooperate with medication according to the doctor's requirements, namely the Diet-Exercise-Medicine pattern. Good emotions have a positive impact on patients' conditions. It is also vital for patients to manage their emotions well under the condition of ensuring good diet habits. Finally, the importance of exercise and medication management is emphasized again in the Exercise-Medicine pattern, indicating that these two aspects are challenging for patients to form habits, which need to be reminded again and paid more attention to it.

## Discussion

According to social cognitive theory (25), a patient's self-management behavior, individual factors, and environmental factors interact. Based on these interaction results, the study puts forward the following suggestions for China outpatients with CKD to improve and support the patient's self-management ability.

For patients, the self-management ability of employed patients in the five dimensions is significantly lower than that of unemployed patients. So, employed patients need to strengthen their awareness and ability in self-management behaviors. Patients should increase interactive communication with medical professional institutions and follow expert opinions. They should maintain a certain exercise frequency and diet habits (24). Exercise intervention is also an effective way to improve CKD patients' self-management ability (26). At last, CKD patients should attach importance to multi-dimensional self-management of disease balance and actively accept systematic self-management services in personality.

For health care providers, medical service providers are an important subject for patients to receive self-management support in the chronic disease care model (CCM) (27), such as hospitals and public health communities (28). This article recommends that medical service providers provide customized intervention programs for patient groups based on chronic kidney disease. Medical service providers should identify patients' demographic characteristics, diagnose patients' self-management patterns, capture and improve the weaknesses of self-management capabilities and implement precise self-management interventions using information technology (29) from social insight (30). For example, for middle-aged and elderly, high-employment, high-education, and medium-expenditure patients with weaker diet behaviors capabilities, hospitals can popularize healthy diet knowledge courses and customize meal lists with the Internet and smart wearable devices to improve patients' cognition and knowledge behavior.

In the social environment, the public generally lacks emotional management (31). Some patients with CKD pay attention to emotional management only in this study. Compared with simply taking medication as prescribed by a doctor, it is more difficult for patients to change their lifestyles, which are hindered by chronic pain and fatigue, interrupted habits, and lack of family support (24). So support from the environmental aspects is added, such as online devices in the patient's home (32).

## Conclusions

To conclude, based on the inductive assumption that patient's self-management need supports from stakeholders, this study investigated and analyzed the cross-sectional data of 131 patients with CKD self-management scale, used the *F*-test to find some demographic characteristics of behavior, and the principal component method to identify the self-management pattern of chronic disease patients among outpatients. The analysis results show that patient with different demographic characteristics has formed four patterns, such as diet behaviors, emotional management, disease cognition, and self-management knowledge to manage their disease. Our study's results help to clarify the self-management behavior patterns and characteristics of different chronic patients, especially for employees, providing a basis for improving the self-management behavior of patients with CKD from three sectors: patients, providers, and society. However, this study only investigated the outpatients of the Nephrology Department and was limited by the scope and number of samples; the results still need more sample tests. This study also has other limitations, such as the lack of detailed patient clustering analysis and more explanations of why the employment status is the most significant, which will be further discussed in subsequent studies.

## Data availability statement

The raw data supporting the conclusions of this article will be made available by the authors, without undue reservation.

## Ethics statement

This study was reviewed and approved by the Ethics Review Committees of West China Hospital (2020-740). All participants provided informed consent before participation in the study.

## Author contributions

XH supervised, reviewed, and edited. YW conceptualized and wrote the first draft. CF, LL, and UK participated in proofreading, reviewing, and editing. FR did formal analysis and editing. XZ reviewed and edited. All authors read and approved the final manuscript.

## Funding

This work was funded by West China Nursing Discipline Development Special Fund Project, Sichuan University (Grant No. HXHL19013) and Natural Science Foundation for the Youth Fund Project of Sichuan University (Grant No. 71904138).

## Conflict of interest

The authors declare that the research was conducted in the absence of any commercial or financial relationships that could be construed as a potential conflict of interest.

## Publisher's note

All claims expressed in this article are solely those of the authors and do not necessarily represent those of their affiliated organizations, or those of the publisher, the editors and the reviewers. Any product that may be evaluated in this article, or claim that may be made by its manufacturer, is not guaranteed or endorsed by the publisher.
