# Supplementary material for: Preferring self-management behavior of patients with chronic kidney disease
Source: Front Public Health. 2022 Dec 2;10:973488. doi: 10.3389/fpubh.2022.973488 (PMC9755185; doi:10.3389/fpubh.2022.973488)
Supplement: Supplementary file 1 [file Table_1.pdf]

Appendix

Table1 F test of self-management behaviour on gender（$x \pm s$）

Items	gender		*F*	*P*
	**male**	**female**		
Medical management	4.34±0.65	4.39±0.60	0.233	0.630
Exercise behaviours	3.94±0.79	4.14±0.68	2.662	0.105
Diet behaviours	4.30±0.72	4.36±0.67	0.268	0.606
Emotional management	4.15±0.78	4.20±1.26	0.059	0.808
Disease cognition	4.28±0.68	4.29±0.69	0.011	0.916
Self-management knowledge	4.37±0.66	4.48±0.64	0.844	0.360

Table 2 Test of self-management behaviour on education level（$x \pm s$）

**Item**	**Education**			***F***	***P***
	**Junior high school and below**	**High school or secondary school**	**University and above**		
Treatment management	3.55±0.44	4.43±0.51	3.96±0.83	1.236	0.309
Exercise behaviours	3.38±0.43	4.14±0.64	3.82±0.63	2.025	0.155
Diet behaviours	3.60±0.71	4.23±0.70	4.05±0.72	1.016	0.378
**Emotional management**	**2.70±0.53**	**4.34±0.43**	**3.80±0.81**	**7.165**	**0.004****
Disease cognition	3.29±0.65	3.95±0.52	3.72±0.73	1.236	0.309
Self-management knowledge	3.69±0.55	4.00±0.63	3.90±0.87	0.207	0.815

Table 3 Test of self-management behaviour on employment status（$x \pm s$）

**Variable**	**Employment status**	***F***	***P***

	Yes	No		
**Treatment management**	**4.28±0.65**	**4.53±0.55**	**1.770**	**0.032***
**Excercise behaviours**	**3.88±0.72**	**4.33±0.70**	**12.019**	**0.001*****
**Diet behaviours**	**4.22±0.68**	**4.52±0.68**	**6.032**	**0.015***
Emotional management	4.15±1.19	4.23±0.79	0.201	0.655
**Disease cognition**	**4.16±0.68**	**4.50±0.66**	**7.370**	**0.008****
Self-management knowledge	4.37±0.63	4.53±0.70	1.897	0.171

Table4 Test of self-management behaviour on disease expenditure（$x \pm s$）

Variable	Disease expenditure (RMB/month)			*F*	*P*
	1000>	[1000~2000]	>2000		
Treatment management	4.47±0.53	4.29±0.65	4.25±0.77	1.570	0.212
Excercise behaviours	4.01±0.77	4.06±0.71	4.08±0.75	0.096	0.909
Diet behaviours	4.40±0.63	4.28±0.73	4.23±0.76	0.631	0.534
**Emotional management**	**4.44±1.25**	**4.00±0.77**	**3.86±097**	**3.507**	**0.033***
**Disease cognition**	**4.43±0.59**	**4.08±0.78**	**4.35±0.67**	**3.513**	**0.033***
Self-management knowledge	4.55±0.56	4.30±073	4.41±0.68	1.997	0.140

Table5 test of self-management behaviour on household income（$x \pm s$）

Variable	Total household income (RMB/month)			*F*	*P*
	5000>	[5000,10000]	>10000		
Treatment management	4.35±0.64	4.27±0.67	4.54±0.51	1.975	0.143
Exercise behaviours	3.98±0.73	4.02±0.78	4.14±0.68	0.461	0.632
Diet behaviours	4.28±0.74	4.23±0.70	4.52±0.57	1.991	1.141
Emotional management	4.11±0.89	4.20±1.42	4.22±0.60	0.127	0.881
Disease cognition	4.37±0.61	4.13±0.79	4.40±0.61	2.130	0.446
Self-management knowledge	4.44±0.66	4.35±0.71	4.53±0.65	0.812	0.446

Table6 test of self-management behaviour on age（$x \pm s$）

Variable	Age (years)			*F*	*P*
	35>	[35,55]	>55		

Variable	Age (years)			*F*	*P*
	35>	[35,55]	>55		
Treatment management	4.30±0.67	4.51±0.57	4.28±0.61	1.797	0.170
Exercise behaviours	3.93±0.84	4.08±0.70	4.15±0.60	0.989	0.375
Diet behaviours	4.22±0.73	4.41±0.64	4.37±0.69	1.000	0.371
Emotional management	4.10±0.82	4.36±1.47	4.04±0.60	1.099	0.336
**Disease cognition**	**4.14±0.77**	**4.50±0.56**	**4.23±0.67**	**3.664**	**0.028***
Self-management knowledge	4.32±0.72	4.57±0.57	4.40±0.63	1.868	0.159
